# PCDHGA10 as a potential prognostic biomarker and correlated with immune infiltration in gastric cancer

**DOI:** 10.3389/fimmu.2024.1500478

**Published:** 2024-12-02

**Authors:** Mingyang Zhong, Zhuoqun Yu, Qianqian Wu, Bing Lu, PingPing Sun, Xiaojing Zhang, Lei Yang, Han Wu

**Affiliations:** ^1^ Department of General Surgery, Medical School of Nantong University, & Department of Gastrointestinal Surgery, Affiliated Hospital of Nantong University, Nantong, Jiangsu, China; ^2^ Clinical and Translational Research Center & Institute of Oncology, Affiliated Hospital of Nantong University, Department of Oncology, Medical School of Nantong University, Nantong, Jiangsu, China

**Keywords:** PCDHGA10, multiplex immunohistochemistry, immunotherapy, prognosis, gastric cancer, tumor microenvironment

## Abstract

**Background:**

Gastric cancer (GC) is one of the most common malignant tumors and is associated with poor prognosis. To improve the prognosis of GC patients, an effective immune-related prognostic biomarker is urgent. Here, we aim to explore the correlation between the expression of procalcitonin gamma subfamily A, 10 (PCDHGA10) and clinicopathological characteristics, especially its relation with tumor-infiltrating immune cells (TILs) in GC.

**Methods:**

The differential mRNA expression of PCDHGA10 between GC tissues and normal gastric mucosa and prognostic potential were assessed from The Cancer Genome Atlas (TCGA). Then, based on tissue microarrays (TMAs) with multiplex immunohistochemistry (mIHC) from GC patients, we statistically assess the correlation between PCDHGA10 protein expression and the clinical profiles and prognosis of the patients. Additionally, with IHC and mIHC, we applied the machine-learning algorithms to evaluate the localization and expression levels of TILs and immune checkpoints in the tumor microenvironment. We analyzed the relationship between PCDHGA10 protein expression and TILs and immune checkpoints.

**Results:**

Through the database and TMA analysis, the expression of PCDHGA10 was significantly higher in GC tissues compared with normal tissues. High PCDHGA10 expression independently predicted poor prognosis in GC. Additionally, elevated PCDHGA10 expression was positively associated with the number of CD8^+^ T cells, CD68^+^ macrophages, Foxp3^+^ T cells, and CD4^+^ T cells in GC tissues and the stromal region. Besides, the expression of PCDHGA10 was positively correlated with immune checkpoints, including CTLA-4, LAG3, and PD-L1.

**Conclusions:**

PCDHGA10 might be a potential prognostic marker and an immunological therapeutic target for GC.

## 1 Introduction

Gastric cancer (GC) is the fifth most prevalent cancer and ranks fourth in terms of mortality rates in the world (1, 2). Due to the difficulty in the diagnosis of early GC, almost up to 70% of patients were in the advanced stage at initial diagnosis (3). The conventional treatment to GC therapy is radical surgery, followed by chemotherapy, which does not lead to acceptable outcomes in these GC patients (4). Therefore, developing innovative treatment strategies for GC is urgent. Over the past few decades, immunotherapy has become one of the most promising cancer treatment strategies (5). However, the tumor immune microenvironment and drug resistance continue to impact immunotherapy’s effectiveness (6). Therefore, an effective immune-related prognostic biomarker is essential for the treatment of GC.

The tumor microenvironment is a unique heterogeneous environment for the tumorigenesis and development of tumors. The growth of tumors and normal tissue homeostasis are both significantly influenced by the bidirectional communication between cells in the tumor microenvironment (TME) (7). Tumor-infiltrating immune cells (TIICs), which are necessary for tumor progression in addition to stromal cells, are a vital component of the TME (8). Accumulating evidence shows that TIICs participate in immunosuppression and progression of GC (9). Immune checkpoint inhibitors (ICIs) are antibody-based therapy that targets immune cells in TME. Blocking the programmed cell death protein 1 (PD-1) or cytotoxic T-lymphocyte antigen 4 (CTLA-4) receptor-ligand interactions could produce favorable therapeutic effects (10). Due to no response to ICIs, only a few patients can benefit from immunotherapeutic interventions. Nevertheless, the existing biomarkers all have advantages and defects when selecting patients who might benefit from immunotherapy.

In the era of tumor immunotherapy, new methods for judging the clinical prognosis and predicting their response to immunotherapy are constantly being developed. These methods are more accurate than traditional methods in screening the beneficiaries of immunotherapy. It is important to choose the most likely group of patients who will benefit from immunotherapy. In comparison with conventional immunohistochemistry (IHC), multiplex IHC (mIHC) can detect the expression of multiple markers simultaneously *in situ*, thereby identifying the phenotype of each cell and cell-to-cell interaction in the tissue, and with quantitative pathology and machine-learning algorithms, highly reproducible statistical data can be obtained (11). Up to now, much research has used mIHC to explore specific immune cells in the tumor immune microenvironment and has contributed to clinical prognosis and efficacy prediction (12).

Procalcitonin gamma subfamily A, 10 (PCDHGA10) is a type I transmembrane protein with 6 or 7 extracellular cadherin repeats, containing 53 genes arranged in strings and belongs to the protocadherin gamma gene cluster. The mutation or the aberrant expression of PCDHGA10 could cause intellectual disability and benign recurrent vertigo (13). The protocadherin gene cluster was verified to participate in regulating tumor progression in lung cancer (14), colorectal cancer (15), and GC (16). Recently, it is reported that PCDHGA10 was highly expressed in lung squamous cell carcinoma, and elevated PCDHGA10 levels exhibited a worse prognosis. Moreover, PCDHGA10 was closely related to tumor immune cell infiltration and immune checkpoints (17). However, there are few studies on the functions of PCDHGA10 in GC.

In the present study, we examined the mRNA level of PCDHGA10 by bioinformatic methods and verified the protein expression of PCDHGA10 via tissue microarrays (TMAs). Then, we applied mIHC to evaluate the relationship between PCDHGA10 and TIICS and immune checkpoints. As an independent predictor of poor outcome, high expression of PCDHGA10 might orchestrate tumor immunity and might be a potential treatment target for GC.

## 2 Materials and methods

### 2.1 Data collection and bioinformatics analysis

The Cancer Genome Atlas (TCGA) (https://portal.gdc.cancer.gov/) datasets were used to collect clinical and RNA-sequence data of patients with GC. Using an online tool Xiantao Academy (https://www.xiantao.love), the mRNA expression of PCDHGA10 from TCGA between the adjacent and tumoral tissues was comparatively analyzed (18). Kaplan-Meier methods were used to assess the survival prognosis of 407 GC samples in TCGA.

### 2.2 Samples collection and TMAs

The TMAs with 195 GC tissues and 70 normal gastric mucosa tissues was prepared at the Department of Clinical Biobank of the Affiliated Hospital of Nantong University from June 2004 to July 2009. A core on the TMAs represents a sample with a diameter of 2 millimeters. All the GC patients underwent radical surgery and did not receive preoperative radiotherapy, chemotherapy, and immunotherapy. From the date of surgery until death or last follow-up, data were collected retrospectively. The study was approved by the Ethics Committee of the Affiliated Hospital of Nantong University.

### 2.3 IHC

TMA slides were dewaxed with xylene and rehydrated with alcohol. TMA slides were then placed in a sodium citrate buffer solution (10 mM, pH 6.0) to repair the antigen through microwave heating. The slides were blocked for 1 h with 5% bovine serum albumin after being treated with 3% H_2_O_2_ for 20 min to eliminate the peroxidase activity. Then rabbit anti-PD-L1 (13684S, Cell Signaling Technology) and mouse anti-CTLA-4 antibodies (NB10064849, NOVUS) were incubated on slides overnight at 4°C. The secondary antibodies were incubated for 2 h and then stained with An EliVision Plus DAB kit (Kit-0015, Maxim Biotechnologie) according to the instructions of the Manufacturers (19). The results of IHC staining were evaluated by the semi-quantitative H-score method with pathologists who were blinded to the patients’ clinical information. The intensity of IHC staining was recorded as 0, 1, 2 and 3, representing no staining, weak staining, moderate staining, and strong staining, respectively. The positivity rate ranges from 0 to 100. The staining intensity and percentage of each sample were evaluated, and the product of each sample was finally calculated as a score ranging from 0 to 300.

### 2.4 mIHC

TMA slides were dewaxed and rehydrated with xylene and alcohol and then were heated in AR6 buffer (210921004, Akoya Bioscience) by microwave to repair the antigen. The blocking buffer (ARD1001EA, Akoya Bioscience) was used for 10 min to block the slides, and the primary and secondary antibodies were added. The mIHC staining was performed after the secondary antibody was added, and then the antigen was repaired through heat induction and cooling. For signal amplification, opal fluorophore-conjugated tyramide signal amplification was used. The nucleus was stained with 4, 6-diamino-2-phenyl indole (DAPI) (F6057, Sigma) and sealed the slides.

The slides were scanned using the Vectra 3.0 Automated Quantitative Pathology Imaging System (PerkinElmer, USA) to detect and measure the positive rate of markers. The cores containing both tumor and stroma were captured with a ×20 Olympus lens objective. With inForm^®^ Cell Analysis software (version 4.1.0, Perkin Elmer), we trained machine-learning algorithms to segment the images into areas of cancerous cells and stromal cells, to segment individual cells by DAPI counterstaining. The pathologists set the threshold of each marker to ensure an accuracy of more than 95%.

This study used the following primary antibodies: rabbit anti-PCDHGA10 (1:50, D1247, Biobyt), rabbit anti-CD11b antibody (1:100, 49420S, CST), rabbit anti-CD8 antibody (1:100, ab83278, Abcam), rabbit anti-CD3 antibody (1:200, 85061S, CST), rabbit anti-CD4 antibody (1:200, ab133616, Abcam), mouse anti-Foxp-3 antibody (1:50, ab20034, Abcam), anti-LAG-3 antibody (1:50, ab52587, Abcam), anti-CD66b antibody (1:500, ab214175, Abcam), anti-cytokeratin antibody (1:8000, orb69073, Biobyt), anti-CD68 antibody (1:500, 797778S, CST). The secondary antibody was Opal™ polymer HRP Ms^+^Rb (211011069, Akoya Bioscience).

### 2.5 Statistical analysis

The association between clinicopathological characteristics and PCDHGA10 was validated using Pearson’s *χ* ^2^. A comparison of mRNA or protein expression between the two groups was performed using the Student’s *t*-test. Cox regression models were used to identify independent prognostic variables. R software (v.4.0.2), GraphPad Prism (v.8.3), SPSS (v.26.0), and X-tile (3.6.1) were used for data analysis. All results with p < 0.05 were considered significant.

## 3 Results

### 3.1 Bioinformatics analysis of PCDHGA10 in GC

**Figure 1:**
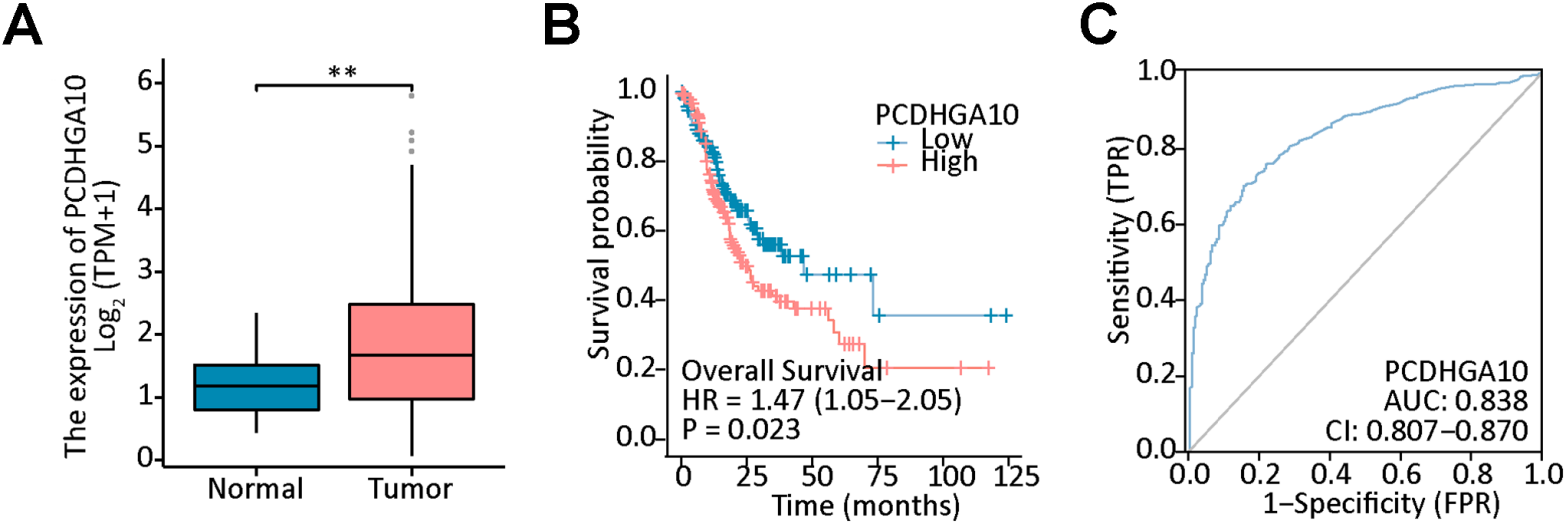
Bioinformatics analysis of PCDHGA10 mRNA expression in gastric cancer (GC). **(A)** The mRNA levels of PCDHGA10 in gastric cancer tissues was higher than that in benign gastric tissues. **(B)** High PCDHGA10 mRNA levels correlated with poor overall survival in GC. **(C)** The receiver operating characteristic curve for PCDHGA10 mRNA levels in GC. ** p < 0.05.

To investigate the role of the PCDHGA10 in GC and peritumoral tissue, the expression of PCDHGA10 was assessed according to the TCGA dataset, which contained 407 GC cases and 32 peritumoral cases. We observed that PCDHGA10 mRNA expression was increased in GC tissues compared to peritumoral tissues (**Figure 1A**, p < 0.001). The level of PCDHGA10 was shown to be up-regulated in GC and related to unfavorable clinical outcomes (**Figure 1B**). The area under the curve (AUC) for PCDHGA10 expression in GC was 0.838 (95% CI = 0.807-0.870) (**Figure 1C**). Therefore, PCDHGA10 was closely related to GC in the PCDHGAs family and may have a vital role in tumorigenesis and development in GC.

### 3.2 PCDHGA10 protein expression in GC

**Figure 2:**
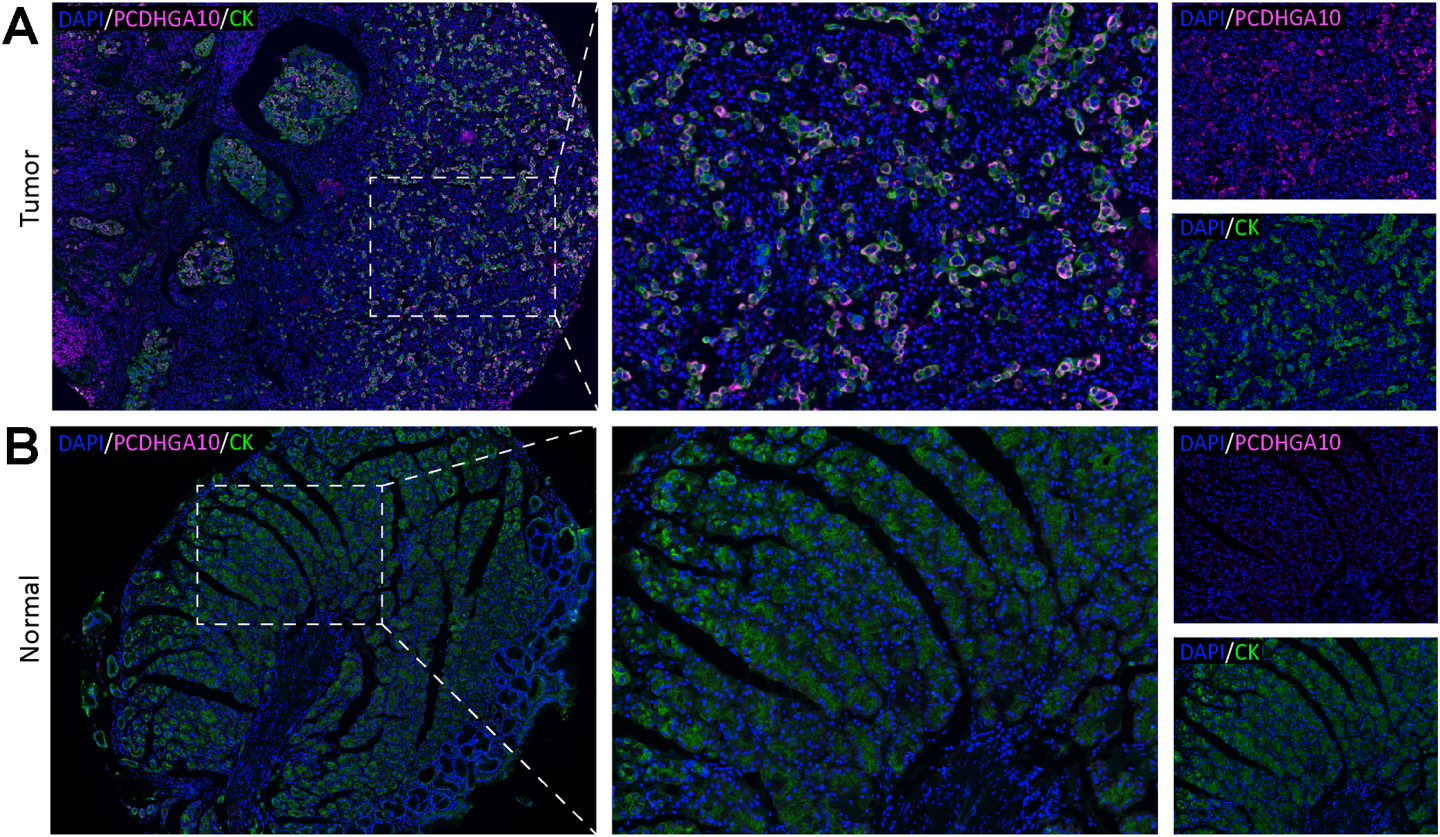
PCDHGA10 protein expression in gastric cancer. PCDHGA10 protein in gastric cancer **(A)** and peritumoral tissues **(B)** with fluorescence multiplex immunohistochemistry. Purplish red: PCDHGA10, green: CK, blue: DAPI. Left column magnification ×40; right two column magnification ×200. DAPI: 4, 6-diamino-2-phenyl indole; CK, cytokeratin.

To confirm the results from the TCGA dataset, we evaluated PCDHGA10 protein expression in GC and para-cancerous tissues using the mIHC technique. Cytokeratin, a marker of epithelial cells, was used to identify tumors and stroma, and nuclei were stained with DAPI. PCDHGA10, primarily found on the cell membrane of GC mucosal epithelial cells, was considerably increased in GC samples, consistent with that of PCDHGA10 mRNA levels (p < 0.05; **Figures 2A, B**).

### 3.3 Association between PCDHGA10 protein expression and GC clinical characteristics

Using the X-tile software, cutoff points of 61.8 were used to categorize patients in TMAs as high or low, including the PCDHGA10-high group (86 cases) and PCDHGA10-low group (109 cases). Following that, the relationship between PCDHGA10 protein expression and clinicopathological characteristics such as gender, age, Laurén categorization, and differentiation, tumor size (T), lymph node metastasis (N), distant metastasis (M), and TNM stage was analyzed, and we observed that PCDHGA10 expression was associated with tumor size (p < 0.05), N (p < 0.05), M (p = 0.01) and TNM staging significantly (p < 0.001; **Supplementary Table 1**).

### 3.4 Prognostic potential of PCDHGA10 protein expression in GC

**Figure 3:**
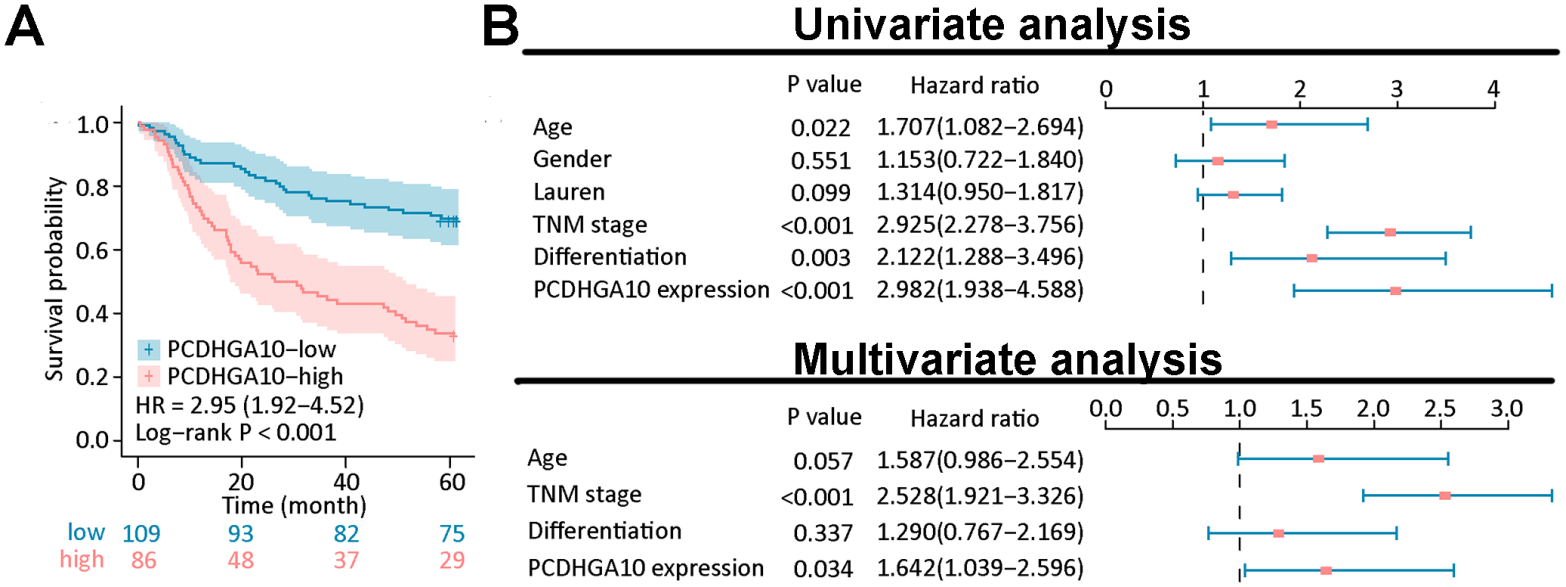
Prognostic analysis of PCDHGA10 protein expression levels. **(A)** Overall survival analysis of PCDHGA10 protein levels based on gastric cancer tissue microarray. **(B)** A forest plot visualizing the univariate and multivariate analysis of PCDHGA10 in gastric cancer.

Kaplan-Meier curve analysis showed that GC patients with high PCDHGA10 protein expression had poor prognosis (**Figure 3A**). As shown in **Figure 3B**, age (p = 0.022), TNM stage (p < 0.0001), differentiation (p = 0.003) and PCDHGA10 expression (p < 0.001) were identified as significant prognostic factors in GC by univariate analysis. Additionally, by the multivariate Cox regression analysis, PCDHGA10 protein expression (p = 0.034), TNM stage (p < 0.001) were found to be an independent risk factor in GC patients.

### 3.5 Relationship between PCDHGA10 protein levels and the tumor microenvironment

**Figure 4:**
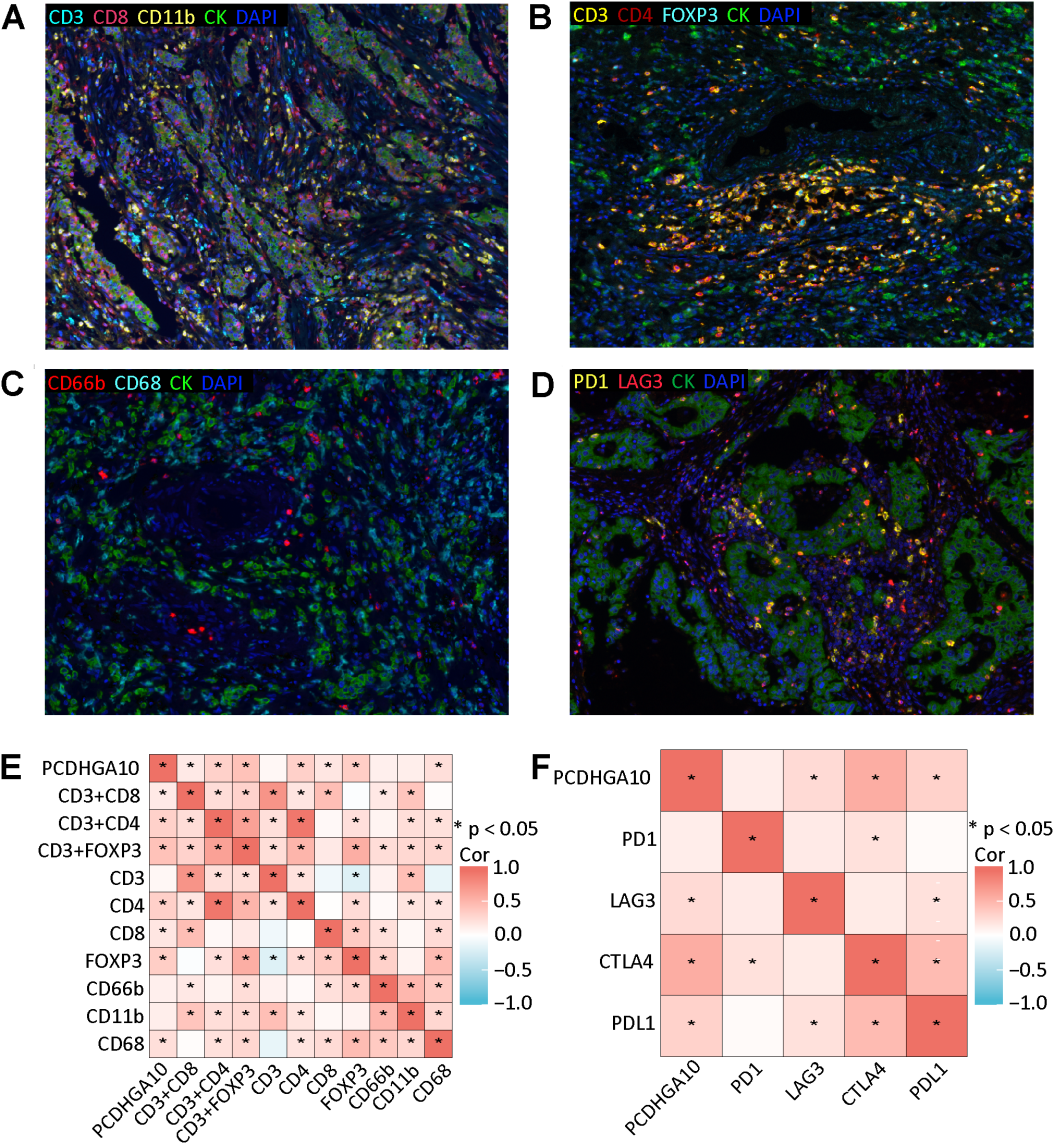
Relationship between PCDHGA10 protein expression and tumor-infiltrating immune cells and immune checkpoints. **(A-C)** Multispectral composite of CD3, CD4, CD8, CD11b, CD66b, CD68, CK and DAPI, magnification ×200. **(D)** Four-color multispectral composite of LAG3, PD1, CK and DAPI, magnification ×200. Green: CK, blue: DAPI. **(E)** Correlation analysis of PCDHGA10 protein expression and tumor-infiltrating immune cells. **(F)** Correlation analysis of PCDHGA10 protein expression and immune checkpoints. DAPI: 4, 6-diamino-2-phenyl indole; CK, cytokeratin. * p < 0.05.

The relationship between the proportion of PCDHGA10 protein expression and the fraction of TIIC in GC tissues was investigated using immune cell-targeted labeling by mIHC. Neutrophils (CD66b^+^), CD8^+^ T cells (CD8^+^), CD4^+^ T cells (CD4^+^), regulatory T (Treg) cells (Foxp3^+^), and macrophages (CD11b^+^, CD68^+^) are the different types of targeted staining indicators. In all samples, immune cells infiltrated to varying degrees, and TIICs were generally found within the tumor stroma (**Figures 4A–C**). Spearman correlation analysis indicated that PCDHGA10 protein level in GC tissues was significantly positively correlated with Foxp3^+^ Treg cells, CD68^+^ macrophages, CD4^+^ T cells, and CD8^+^ T cells (**Figure 4E**). However, a significant association between PCDHGA10 expression and CD66b^+^ neutrophils is not observed in GC tissues.

Then we verified the relationship between PCDHGA10 and immune checkpoints by mIHC and IHC. mIHC staining showed that PD-1 and LAG3 were mainly localized in tumor mesenchyme (**Figure 4D**). Positive correlations were observed between PCDHGA10 protein expression and LAG3. IHC staining showed that CTLA4 and PD-L1 were also expressed to varying degrees in GC tissues (**Supplementary Figure S1**). Statistical analysis revealed that the protein presentation of PCDHGA10 was significantly correlated with the protein presentation of CTLA4 and PD-L1. However, no significant association was identified with regard to PD-1 protein expression (**Figure 4F**).

## 4 Discussion

The present study showed that PCDHGA10 mRNA was considerably more expressed in tumor tissues than in normal tissues using the TCGA datasets. In addition, PCDHGA10 was identified as an independent poor prognosis factor. Moreover, it was found that PCDHGA10 expression is associated with clinicopathological features and involved in immune cell infiltration, thus making it a promising target for cancer immunotherapy.

The interactions between tumor and immune cells in the tumor immune microenvironment (TIME) determine the trend of anti-tumor or pro-tumor immunity (9). Tumors appear to evade immune surveillance by gradually shaping the TIME into an immunosuppressive state through recruiting tumor-promoting immune cells, and the balance between pro- and anti-tumor inflammatory mediators may determine tumor progression (20, 21). Our results revealed that PCDHGA10 expression positively correlated with Foxp3^+^ T cells. Foxp3^+^ is a specific surface marker for Treg cells, which play a key role in maintaining immune homeostasis and peripheral tolerance (22, 23). Additionally, Tregs inhibit anti-tumor immune response by producing immunosuppressive cytokines, such as TGF-β, IL-10, and IL-35 in the TIME (24). Up to now, many studies have demonstrated the extensive infiltration of Tregs in malignant tumors, including gastric cancer, is associated with poor prognosis (25). In addition, we found that the expression of PCDHGA10 was related to CD68^+^ tumor-associated macrophages (TAMs). TAMs are divided into anti-tumor M1-type macrophages and tumor-promoting M2-type macrophages. It is accepted that CD68 and F4/80 are the markers of the M2-type TAMs (26). M2 macrophages are usually the dominant cells in TAMs and secrete immunosuppressive cytokines to promote tumor immune escape (27, 28). Chen et al.*’s* reported that GC patients with a high density of CD68^+^ TAMs tended to have a bad prognosis (29). These results established a link between PCDHGA10 protein expression and TIICS in GC.

The emergence of ICIs, mainly including PD-1/PD-L1, LAG-3, and CTLA-4 monoclonal antibodies (mAbs), has shaped the therapeutic landscape of some types of cancers (30). It is reported that some cancer patients’ improved survival outcomes are largely due to the improved control of systemic disease provided by ICIs (31, 32). s. Disrupting co-inhibitory signaling pathways enhances clinical outcomes in cancer patients (33, 34). Currently, anti-CTLA-4 agents such as Ipilimumab and Tremelimumab are broadly applied as therapeutic agents in clinical studies of different cancers (35, 36). In a randomized, phase III trial, compared to chemotherapy alone, the PD-1 inhibitor Nivolumab combined with chemotherapy showed superior overall survival (OS) and progression-free survival (PFS) in previously untreated patients with advanced gastric adenocarcinoma (37). Despite immunotherapy having markedly improved the survival rate of patients in certain tumor types, not all patients benefit from checkpoint blockade, and some suffer from notable immunotoxicities (38, 39). Thus, it is crucial to identify potential biomarkers suitable for screening the population who might benefit from immunotherapy (40–42). In the present study, we found that the protein expression levels of PCDHGA10 were correlated with PD-L1, CTLA-4, and LAG-3 with mIHC. Currently, available evidence indicates that PD-L1, tumor mutational burden (TMB), and microsatellite instability (MSI)/mismatched repair-deficient (MMR) have been acknowledged for screening the population in whom immunotherapy is effective of immune drugs (43–45). So, combined with the above-mentioned effective immunotherapy predictors, PCDHGA10 might be a biomarker that predicts immunotherapy responses in GC.

There are several limitations in this study. First and foremost, our study was retrospective research, and additional prospective research is required to strengthen our conclusions. Additionally, the interaction mechanism between PCDHGA10 and immune cells and immune checkpoints in GC needs further experimental verification.

- Our results show that PCDHGA10 is up-regulated dramatically in GC and is an independent prognostic factor. It is revealed that PCDHGA10 is correlated with TIICs as well as immune checkpoint expression in GC. Thus, PCDHGA10 might be a potential biomarker for predicting GC prognosis and provides a perspective on immunotherapeutic strategies for treating GC.

## Data Availability

The data presented in the study are included in the article/**Supplementary Material**, further inquiries can be directed to the corresponding authors.

## Supplementary material

Supplementary Figure 1 Quantified protein expression using the immunohistochemistry score (intensity: brown, intense staining; orange, moderate staining; yellow, weak staining; and blue, no staining. A1: CTLA4 in cancer tissues. A2: CTLA4 expression in cancer cells was scored using software. B3: CTLA4 expression in TILs was scored using software. Magnification ×200.
