# Supplementary figures and images for: PCDHGA10 as a potential prognostic biomarker and correlated with immune infiltration in gastric cancer
Source: Front Immunol. 2024 Dec 2;15:1500478. doi: 10.3389/fimmu.2024.1500478 (PMC11647002; doi:10.3389/fimmu.2024.1500478)

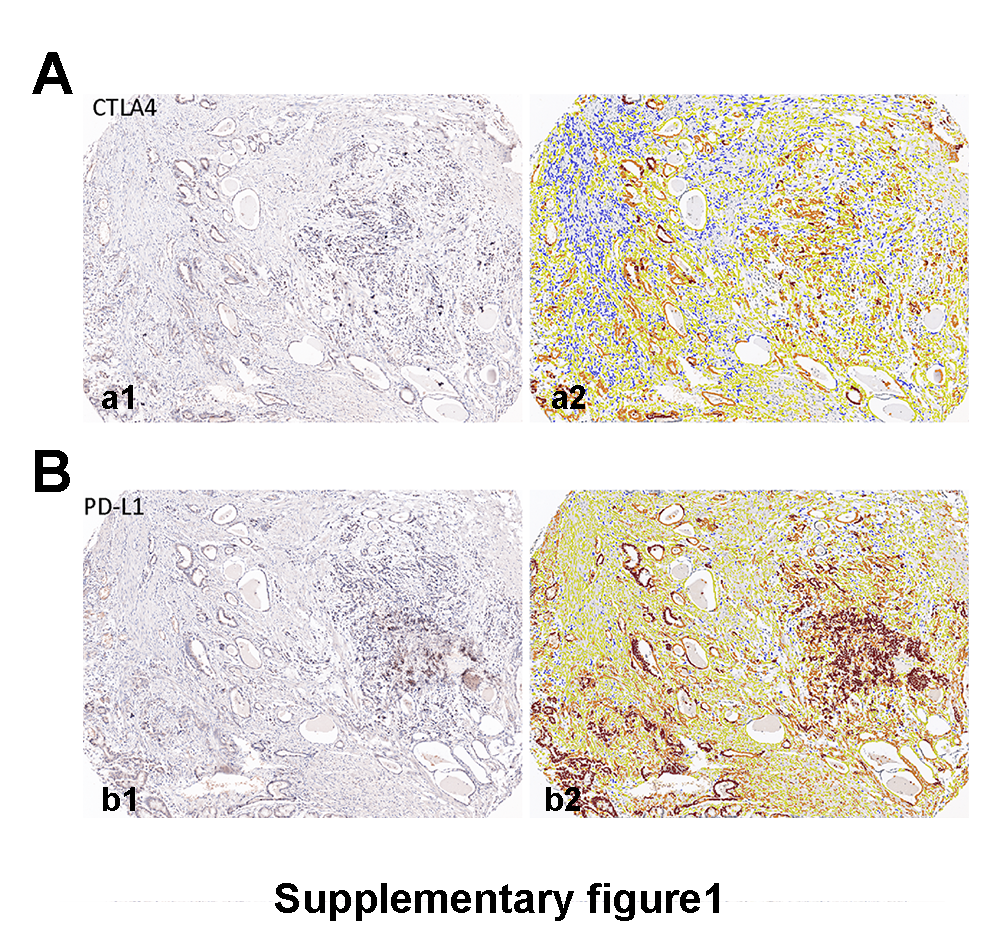

Supplement: Supplementary Figure 1 — Quantified protein expression using the immunohistochemistry score (intensity: brown, intense staining; orange, moderate staining; yellow, weak staining; and blue, no staining. A1: CTLA4 in cancer tissues. A2: CTLA4 expression in cancer cells was scored using software. B3: CTLA4 expression in TILs was scored using software. Magnification ×200. [file Image1.tif]
