# Supplementary material for: PCDHGA10 as a potential prognostic biomarker and correlated with immune infiltration in gastric cancer
Source: Front Immunol. 2024 Dec 2;15:1500478. doi: 10.3389/fimmu.2024.1500478 (PMC11647002; doi:10.3389/fimmu.2024.1500478)
Supplement: Supplementary file 2 [file Table1.docx]

**Resubmission statement**

Dera Editor,

We submitted a manuscript entitled “PCDHGA10 Correlates with Tumor Immunity and Facilitates Unfavorable Prognosis of Gastric Cancer” (MS ID: 1414847) to *Frontiers in Oncology, section Pharmacology of Anti-Cancer Drugs* several months ago. But we felt that our research did not incorrectly submitted and the article is not well written enough. So after carefully thinking, we decided to withdraw the manuscript with great pity. After our carefully discussion, we reorganized the structure of the article and polished the literal expression and grammar to better present our research content.

Yours sincerely,

Professor Han Wu

Corresponding author
